# Implementation of a High-Accuracy Neural Network-Based Pupil Detection System for Real-Time and Real-World Applications

**DOI:** 10.3390/s24082548

**Published:** 2024-04-16

**Authors:** Gabriel Bonteanu, Petronela Bonteanu, Arcadie Cracan, Radu Gabriel Bozomitu

**Affiliations:** 1 Fundamentals of Electronics Department, “Gheorghe Asachi” Technical University of Iasi, 700050 Iasi, Romania; gbonteanu@etti.tuiasi.ro (G.B.); acracan@etti.tuiasi.ro (A.C.); 2 Telecommunications and IT Department, “Gheorghe Asachi” Technical University of Iasi, 700050 Iasi, Romania; petronela.bonteanu@student.tuiasi.ro

**Keywords:** artificial intelligence, pupil detection, neural networks, classifier, real-time

## Abstract

In this paper, the implementation of a new pupil detection system based on artificial intelligence techniques suitable for real-time and real-word applications is presented. The proposed AI-based pupil detection system uses a classifier implemented with slim-type neural networks, with its classes being defined according to the possible positions of the pupil within the eye image. In order to reduce the complexity of the neural network, a new parallel architecture is used in which two independent classifiers deliver the pupil center coordinates. The training, testing, and validation of the proposed system were performed using almost 40,000 eye images with a resolution of 320 × 240 pixels and coming from 20 different databases, with a high degree of generality. The experimental results show a detection rate of 96.29% at five pixels with a standard deviation of 3.38 pixels for all eye images from all databases and a processing speed of 100 frames/s. These results indicate both high accuracy and high processing speed, and they allow us to use the proposed solution for different real-time applications in variable and non-uniform lighting conditions, in fields such as assistive technology to communicate with neuromotor-disabled patients by using eye typing, in computer gaming, and in the automotive industry for increasing traffic safety by monitoring the driver’s cognitive state.

## 1. Introduction

In an age where communication has become the core of human connectivity, where information flies at unimaginable speeds and our digital world evolves at an astonishing rate, the concept of communicating is more vital than ever. In an era when electronic devices are part of our daily lives and assist us in every aspect of life, the development of efficient and intuitive human–machine interfaces has become a crucial priority. However, at the same time, we can say with confidence that we live in an era when our gaze, this subtle and expressive tool of our communication, may hold the key to a truly revolutionary human-machine interface.

Eye tracking systems have evolved significantly in recent decades, transforming from research and cognitive science tools to advanced technologies with the potential to redefine the way we communicate with our electronic devices. These immersive systems enable the precise monitoring of eye movements, providing a direct window into our intentions, attention, and emotions. In today’s context, in which our gadgets react to gestures, voice commands, and touch commands, eye tracking is the element that completes this arsenal, allowing devices to understand not only what we say but also what we feel and look at.

Over the years, techniques for pupil center detection have been implemented using classical image processing methods or using different AI-based architectures.

Many achievements have represented significant advances in the field of gaze detection using traditional image processing methods, ensuring accuracy and robustness in a variety of scenarios. The Starburst algorithm is an iris tracking technique that is based on the identification of iridescence using characteristic radial lines and corners of the iris [1]. The projection algorithm uses the projection of pixels on the *x* and *y* axes to detect the iris and pupil in images [2]. Algorithms based on contour analysis detect the iris and pupil by extracting their shapes from images by following edges [3]. The integral projection method is based on the projection of the sum of pixels in the horizontal and vertical directions to locate the iris and pupil [4]. Algorithms using the Gaussian model focus on modeling the distribution of pixels in the image to estimate iris parameters [5]. The circular Hough transform [6] and elliptical Hough transform [7] are used to detect circles in images, including the iris and pupil. Algorithms based on least squares ellipse fitting (LSFE) detect the iris and pupil by fitting an ellipse to their outline [8].

To these are added robust, complex algorithms for pupil detection in real-life scenarios. Algorithms such as ExCuSe [9], ElSe [10], and PuReST [11] are examples of complex approaches that successfully address environmental variables to ensure accurate pupil detection in real-world scenarios, but their performance still leaves something to be desired in variable lighting conditions.

The main disadvantage of pupil detection algorithms (PDAs) based on classical image processing is the fact that these algorithms do not work properly in nonuniform and variable lighting conditions specific to real-time applications.

Although gaze detection algorithms based on classic image processing methods have represented a significant step in the development of human-machine interfaces, it is important to mention that solutions based on artificial intelligence (AI) are increasingly preferred. AI-based architectures, such as deep neural networks, have demonstrated a remarkable ability to interpret and understand the complexity and subtleties of human behavior, allowing for the development of more sophisticated and adaptable gaze detection systems [12]. These AI solutions offer significant potential for personalizing human-computer interactivity and increasing the efficiency of these interfaces.

Eye tracking systems based on artificial intelligence are used to monitor and understand the visual behavior of individuals. These technologies find applications in research, human-machine interfaces, medicine, advertising, web design, computer gaming, military applications, driver fatigue detection, and more. Over the years, various techniques and methods have been developed to improve the accuracy and efficiency of these systems.

One of the most important developments was the application of deep neural networks (deep learning) for eye tracking. Convolutional models (CNN) and recurrent networks (RNN) are used to detect key features of the eyes and predict the fixation point. CNNs are trained to recognize eye features such as the iris and sclera. By tracking these features, the system can determine gaze direction [13]. Approaches for estimating gaze direction in unprotected environments, such as outdoors, have also been proposed.

Other techniques for image-based pupil detection combine convolutional neural network architectures with different refinement methods to increase detection accuracy, as in the case of PupilNet v2.0, illustrated in [14].

RNNs can account for the time evolution of the gaze, making them suitable for gaze tracking tasks [15]. Techniques that use synthesized images to develop high-quality representations of gaze direction have been presented.

Instead of relying on only a single camera, gaze-tracking systems can use multiple cameras to improve accuracy and create 3D gaze images [16]. One can continuously estimate gaze in the context of 3D displays, using multiple cameras to obtain an accurate estimate of a three-dimensional gaze. Gaze data are collected and processed with AI algorithms to obtain accurate estimates of gaze and gaze depth.

Another important goal is to allow for the implicit calibration of gaze tracking systems. This means that there is no need for the user to perform long and tedious calibrations to start using the system. GazeTutor, proposed in [17], is a real-time gaze tracking system that can be used on shared displays without prior calibration.

With increasing concerns about ethics and data privacy, researchers have also focused on developing eye tracking models that can explain and justify their decisions [18], which can be important for users’ understanding and trust in the system. The use of RNNs to estimate gaze fixation points is explored, and how explanations for these estimates can be generated is discussed.

Deep Gaze Estimation [19] is an approach that uses deep convolutional neural networks (D-CNNs) to estimate gaze direction. Deep learning models are trained on large datasets to learn key eye and face features that indicate gaze direction.

In the Eye Movement Analysis with Machine Learning [20] approach, eye movement data (such as gaze velocity and trajectory) are recorded and analyzed with machine learning algorithms to infer the user’s intentions and interests.

Another method combines information from eye tracking with head tracking to obtain more accurate estimates of gaze direction [21]. AI algorithms can be used to combine this information in an efficient way.

Instead of using invasive hardware devices (such as sensor glasses), the Non-Intrusive Gaze Tracking approach uses AI techniques that rely on face video data to track the gaze [22].

Gaze Gesture Recognition technology uses AI to recognize eye gestures, such as blinking and the opening or closing of the eyelids, to interpret the user’s intentions [23].

Each of these techniques relies on AI approaches to detect and track the gaze. It is important to note that this field is constantly evolving, and researchers are constantly developing new methods and technologies to improve the accuracy and efficiency of eye tracking.

In light of rapid advances in artificial intelligence, this paper proposes an innovative solution for pupil detection by means of a neural network-based classifier. This promising technology can be used for a variety of other vital applications. For example, in the field of medical diagnosis, AI classifiers have revolutionized the interpretation of medical images [24]. In sound filtering, AI-based technologies have proven essential for noise reduction in audio recording systems [25]. In natural language recognition, AI methods enable meaningful conversations between humans and machines [26]. In the field of fraud detection, AI algorithms assist in the detection of fraudulent behaviors in real time [27]. Sentiment analysis also uses AI to assess and interpret human emotions in text and speech [28]. In self-driving assistance, autonomous vehicles use AI classifiers for traffic navigation and decision-making [29]. Image classification enjoys increased accuracy thanks to AI technologies [30]. Finally, signal segmentation becomes more efficient through AI algorithms, thus improving data analysis processes [31]. This work aims to contribute to the development of this innovative technology and explore its potential in the context of pupil detection, as part of a larger effort to bring significant benefits to society in line with current advances in AI.

In the continuation of this paper, we will explore in detail the implementation of a pupil detection system using an AI classifier in low-resolution images, addressing the key technical and conceptual issues underlying this procedure. We will also examine the challenges in extending this solution to high-resolution images, analyzing the difficulties in processing and interpreting the complex details of these images. In this context, we will propose an efficient solution in terms of the number of neurons required to handle high-resolution images, thus providing an optimized approach.

The proposed solution is based on a new parallel architecture using two convolutional slim neural networks in order to detect the two pupil center coordinates. The main advantages of the proposed solution consist of high accuracy, a high frame processing rate, low architecture complexity, and high reliability in non-uniform and variable lighting conditions, making it suitable for real-time and real-world applications. We will discuss how to preprocess the images, train the classifier, and evaluate its performance in terms of accurate pupil center detection.

To validate and evaluate the efficiency of the proposed CNN architecture, we will present the results obtained from the training and testing process on an extensive dataset, including ~40,000 eye images, collected from four distinct sources.

Finally, this paper will conclude with a comprehensive discussion of the possible applications of the proposed solution in various fields, thus opening new perspectives for the use of this technology in a wide spectrum of contexts and applications.

The rest of this paper is organized as follows: Section 2 illustrates the development of new pupil detection solutions using neural network-based classifiers. In this section, an architecture for pupil center detection suitable for low-resolution eye images is presented, as is a novel AI-based pupil detection system that uses two classifiers implemented with slim-type neural networks capable of processing high-resolution eye images. In Section 3, the training of the proposed system and the experimental results are presented. In Section 4, some discussions regarding the performance of the proposed solution are presented, and Section 5 outlines the conclusions.

## 2. Development of New Pupil Detection Solutions Using Neural Network-Based Classifiers

### 2.1. Investigating a Solution for Pupil Center Detection in Low-Resolution Eye Images

**Figure 1:**
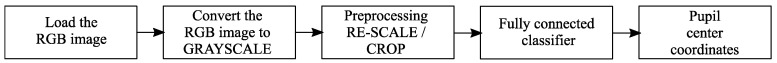
The structure of the pupil center detection system suitable for low-resolution eye images.

In Figure 1, the structure of the pupil center detection solution suitable for low-resolution eye images is presented. The images provided by the video camera are converted to grayscale, resized to a size of 20 × 15 pixels, and then processed by a fully connected neural network-based classifier to identify the coordinates of the pupil center.

The implementation of the classifier on such low-resolution images will be exemplified first, and in Section 2.2 it will be presented the development of a classifier that is able to process images of a significantly higher resolution, 320 pixels by 240 pixels, showing the necessary changes that need to be made to the application.

The first step of a pupil center detection application using a classifier is, of course, data collection and labeling. The images we used come from the following sources: images acquired using a device developed by our research team [7], images from the public Casia Iris Lamp database [32], public images provided by Świrski in [33], and images made public by the authors of the ExCuSe algorithm [34].

The first dataset (DB1) consists of 400 infrared eye images with a resolution of 640 × 480 pixels, with eye pupils of different shapes placed in different positions on the sclera and multiple corneal reflections captured with the head-mounted eye tracking interface developed by our team. The second dataset (DB2) includes 400 infrared eye images with a resolution of 640 × 480 pixels from the publicly available database Casia-Iris-Lamp [32]. The third dataset (DB3), provided by Świrski, contains 600 eye images with a resolution of 620 × 460 pixels, highly off-axis, with eyelashes as presented in [33], obtained as a uniformly random subset of left and right eye videos from two people. The 17 databases used by the ExCuSe algorithm [9] and Else algorithm [10], noted in DSI-DSXVII, include over 38,000 hand-labeled eye images from real-world tasks with a resolution of 384 × 288 pixels [34]. The eye images from the DB1, DB2, and DB3 databases have been resized to a resolution of 320 × 240 pixels, and the eye images from the DSI-DSXVII databases have been cropped to the same size. These images were manually labeled to indicate the pupil center coordinates.

**Figure 2:**
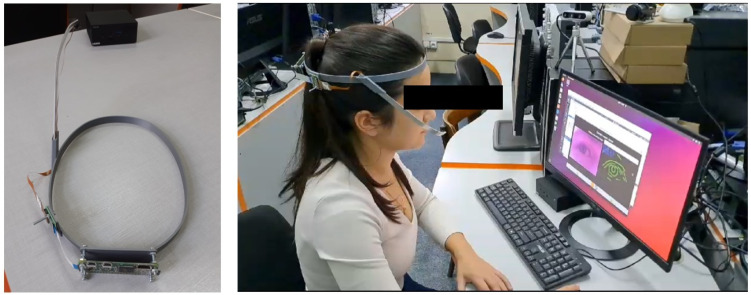
Testing of the proposed eye tracking system in laboratory conditions.

In Figure 2, the eye tracker system implemented by our team is illustrated. It includes hardware and software components. The hardware component consists of a head-mounted device, an infrared video camera (Spy Camera HD 5MPx Flex NoIR for Raspberry Pi) mounted on a special frame, and a processing unit (represented by a computer) used for eye image processing. The software component is represented by the proposed AI-based pupil detection solution. This system has been used in order to acquire eye images from the DB1 database.

The second stage is data preprocessing (images in the dataset may require preprocessing to ensure they are of uniform size and quality). The images were processed so that, regardless of the source, they have the same resolution of 20 pixels by 15 pixels.

**Figure 3:**
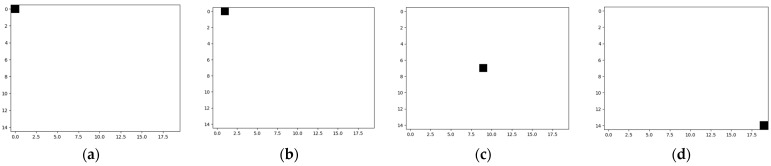
The expected operation of the neural network-based classifier: (**a**) Class 1 corresponds to those eye images in which the center of the pupil is in the upper left corner of the image; (**b**) Class 2 corresponds to images in which the center of the pupil is on the first line and the second column of the image; (**c**) Class 150 corresponds to images in which the center of the pupil corresponds to the center of the image; (**d**) Class 300 corresponds to images in which the center of the pupil corresponds to the bottom right corner of the eye image.

Next, it is necessary to define the classes of the fully connected neural network-based classifier used in the system suitable for low-resolution eye images, illustrated in Figure 1. In this case, a number of classes equal to the number of pixels in the image is necessary (300 classes for the considered eye image resolution). Thus, Class 1 corresponds to those eye images in which the center of the pupil is in the upper left corner of the image, Class 2 to images in which the center of the pupil is in the first line and the second column of the image, and so on, with the last class corresponding to images in which the center of the pupil is in the lower right corner. Some such classes are exemplified in Figure 3 by illustrating the desired response of the neural network.

The operation expected from such a classifier implemented with the neural network is the following: one and only one of the neurons on the last layer will be activated, corresponding to the class to which the image applied to the input of the network belongs.

**Figure 4:**
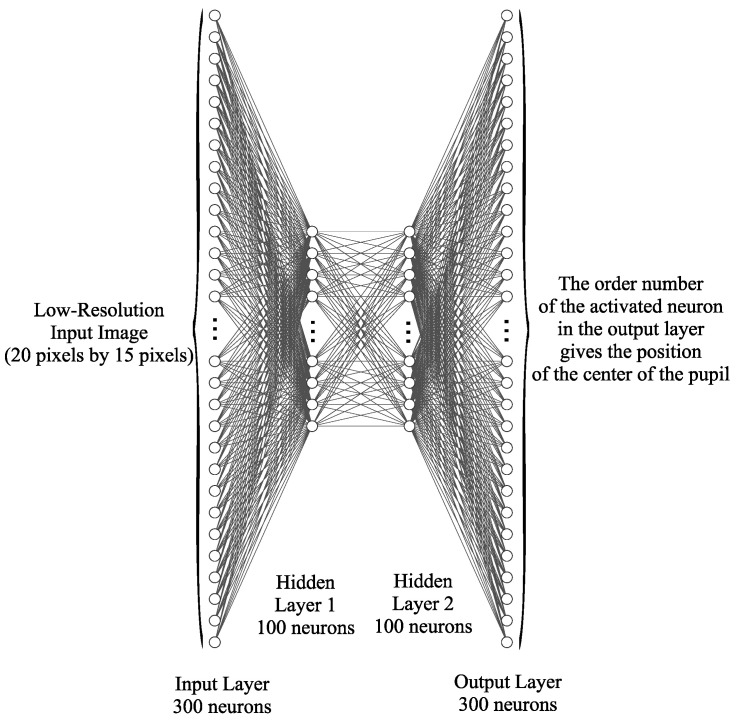
The architecture of the fully connected neural network-based classifier used by the pupil center detection system is suitable for low-resolution eye images.

Due to the reduced number of pixels in the input images, the neural network can be implemented with layers of fully connected ReLU neurons. An architecture with four layers of neurons was used for implementation, as Figure 4 shows: an input layer containing 300 neurons that encodes the normalized gray intensities of each image pixel, two hidden layers of 100 neurons each, and an output layer that contains a number of neurons equal to the number of possible classes (actually the number of pixels in the image).

To calculate the total number of these neural network parameters, we considered the connections between neurons and each parameter associated with them. Thus, this neural network has a total of 70,500 parameters (weights and biases). The relationship between the amount of training data and the number of parameters of a neural network is essential to achieving good performance and avoiding problems such as overfitting or underfitting. In the case of a small amount of training data and a neural network with a large number of parameters, there is an increased risk of overtraining. The network might learn the training data in detail and not generalize correctly to new, unseen data. In order to prevent overfitting, which occurs when the model fits the training data too well and does not generalize correctly to new, unseen data, the regularization technique was used. Regularization can prevent extreme values of parameters in the neural network, which can lead to instability and inefficient learning. By adding a regularization term in the cost function, the uncontrolled growth of the parameters was limited.

The cross-entropy cost function was preferred in training the neural network for the classification problem over the quadratic cost function because it is the natural choice for such a task, due to the fact that it effectively quantifies the discrepancy between the true distribution of classes and the distribution predicted by the model [35]. In the case of cross-entropy, the gradient of the error with respect to the model parameters is more stable compared to the squared error, especially when the predicted probabilities are close to 0 or 1. This makes the training more efficient and less prone to slow convergence.

The training of the neural network was performed with stochastic gradient descent (SGD); this involves optimizing the parameters of the network during training using a gradient descent approach. The main difference between SGD and traditional gradient descent (GD) is that SGD uses a random subset (mini-batch) of training examples to compute the gradient and update the model parameters at each step.

The first step for training the neural network with SGD was initialization. It started by initializing the neural network parameters (weights and biases) with random or pre-trained values. Next, during mini-batch selection, the training set was divided into small-sized mini-batches. The size of these mini-lots is one of the hyperparameters that can be adjusted; in this case, size 10 was used. A workout consisted of a number of epochs, with an epoch being a complete run through the training set. At each epoch, the training data was randomly shuffled and then iterated through a sequence of mini-batches. For each mini-batch, the gradient of the cost function was calculated with respect to the network parameters. This involved calculating the partial derivative for each model parameter. After calculating the gradient for a mini-batch, the model parameters were updated using the gradient descent formula:

(1) new_parameter=old_parameter−learning_rate·gradient

where “*old_parameter*” represents the value of the parameter in the previous step, “*learning_rate*” is a hyperparameter that controls how large the update step is, and “*gradient*” is the gradient calculated for that mini-batch.

During training, model performance was monitored on a separate validation set to assess how well it generalizes to new data. Training was stopped after a fixed number of epochs or after performance on the validation set no longer improved. After training the model, its performance was evaluated on an independent test set to obtain an objective assessment of the model’s quality.

The SGD training process was repeated until the model achieved the desired performance. Adjusting parameters such as learning rate and mini-batch size was necessary to obtain the best results.

In general, the hyperparameter tuning strategy was to start the training with a higher-value learning rate in a relatively large number of epochs (hundreds) until a plateau was reached in terms of classifier accuracy, after which the learning rate was progressively reduced by an order of magnitude in a significantly reduced number of epochs (tens) until the accuracy stabilized again. The high number of initial training epochs was justified by the fact that the parameters of the network, weights and biases, were randomly initialized while the values resulting from pre-training were used in the subsequent training phases.

The available eye image data (~40,000 eye images from 20 different databases) were organized as follows: 80% were used for the actual training set, 10% comprised the validation dataset, and the remaining 10% comprised the test dataset.

The test results of this version of the classifier indicated an accuracy of 80% for the exact identification of the pupil center, while the detection rate at 2 pixels reached 100%.

### 2.2. A Novel Pupil Detection System Based on Neural Network Classifiers Suitable for High-Resolution Eye Images

Expanding the technical solution previously presented regarding the ability to process low-resolution images, 20 pixels by 15 pixels, to high-resolution images, 320 pixels by 240 pixels, is not physically feasible. This is primarily due to the huge number of neurons required for the output layer. Thus, for an image with a geometry of 320 pixels horizontally and 240 pixels vertically, the number of pixels is 76,800, which is equal to the number of possibilities for placing the center of the pupil. A similar architecture for a neural network would mean two hidden layers, each with 25,600 neurons. The number of parameters in such a network would be 3,587,128,000, the impossibility of implementing and training being obvious.

**Figure 5:**
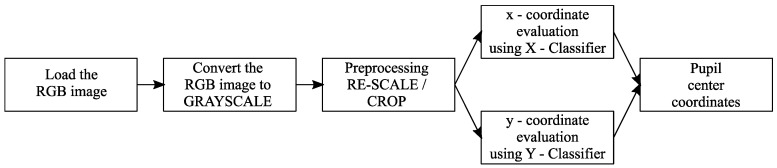
The structure of the pupil center detection system suitable for high-resolution eye images.

In what follows, a solution for implementing a pupil center detection system for high-resolution eye images (320 × 240 pixels) is presented in an efficient way from the point of view of resource consumption, with the structure of the proposed system being illustrated in Figure 5. The images provided by the video camera are converted to grayscale, resized to a size of 320 × 240 pixels, and then processed by two slim neural network classifiers to identify the coordinates of the pupil center.

The first step to simplifying the classifier based on neural networks capable of processing high-resolution images is to reduce the number of outputs. As seen in the Section 2.1, a simple and straightforward implementation involves having a number of outputs equal to the number of pixels in the input image, *h∙v*, where *h* is the image horizontal size in pixels and *v* is the number of pixels on the vertical. This happens because the center of the pupil can be in any of the pixels of the image.

A significant reduction in the number of outputs would result if the proposed approach provided separately the two pupil center coordinates (*x* and *y*) by using two distinct neural network classifiers, respectively. Cumulatively, for the two neural networks, we will have the total number of neurons in the last layer given by the sum of the two dimensions of the input image, *h* + *v*, a value significantly lower than the product of the two parameters:

(2) h+v≪h·v

Thus, an architecture of two neural networks will be used to process the information in parallel. Both will receive the intensities of the image pixels as inputs (so we can consider that the input neuron layer is common), while the rest of the neuron layers will be independent in the sense that the first neural network will process the information to deliver the *x-coordinate* of the pupil center, while the second neural network will independently process the information to deliver the *y-coordinate*.

Although we are apparently dealing with a doubling of the number of neural networks, there will be a significant reduction in the number of neurons used overall because the first classifier will only have *h* outputs (*h* ≪ *h∙v*), while the second classifier will only have *v* outputs (*v* ≪ *h∙v*). The reduced number of outputs of the two classifiers, of course, will imply a reduced number of neurons for the hidden layers of the network, thus leading to massive savings in resources compared to the straightforward implementation of the classifier with *h∙v* outputs. Therefore, the main advantage of the proposed architecture consists of its reduced complexity, which includes two slim neural network classifiers. Another important advantage of the proposed solution is that it precisely detects the coordinates of the pupil center, unlike other similar AI-based implementations, which only detect the area where the pupil is located, using an additional processing stage (computationally expensive) for the precise detection of the pupil center, as illustrated by PupilNet v2.0 in [14].

**Figure 6:**
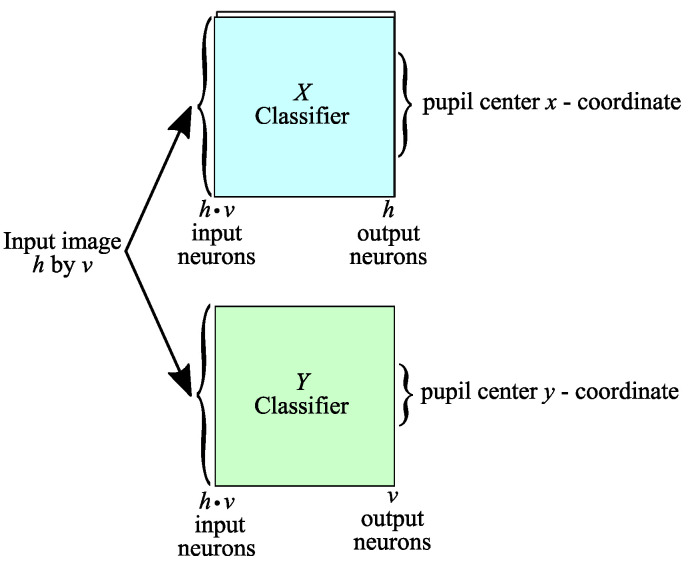
Architecture of the classifier implemented with two slim neural networks used by the pupil center detection system suitable for high-resolution eye images.

The principle exemplified above is illustrated in Figure 6 for an image of size *h* pixels by *v* pixels. The grayscale intensities of the eye image pixels are provided to both classifiers. They process the information independently, and each provides a coordinate of the center of the pupil. The *x-coordinate* of the center of the pupil results from the activation of *h* neurons in the output layer of the *X* Classifier, while the *y-coordinate* of the pupil center results from the activation of *v* neurons in the output layer of the *Y* Classifier.

In order to make the use of resources even more efficient, a convolutional architecture was chosen for the two independent networks that implement the classifier for the *x-coordinate* and the classifier for the *y-coordinate*, respectively.

The convolutional neural network (CNN) architecture offers several advantages over a fully connected neural network (FCN) architecture when used in image and spatial data processing problems. CNNs are designed to extract hierarchical features from images or spatial data. They use convolution layers to detect simple features such as edges and textures, and then deeper layers to combine these simple features into more complex and abstract features. This hierarchical approach is effective at identifying meaningful details from the input data. Also, CNNs use pooling or subsampling layers to reduce the dimensionality of the data, which reduces the number of parameters and computations required in the network. This makes CNNs more efficient in training and inference. Further, CNNs use convolution filters that are shared across the entire image. This allows the parameters to be reused to detect the same features in different locations of the image. In a fully connected network, each neuron in a layer has its own parameters, which leads to an exponential increase in the number of parameters depending on the size of the input. Due to the use of convolutions and pooling, CNNs are better able to achieve translation-invariant object recognition; that is, they can identify objects in different positions in the image without the need for separate training for each position.

CNNs are optimized for the efficient processing of images and other spatial data. By using convolution filters and pooling layers, intensive computations can be performed more efficiently. Also, CNNs have achieved impressive performance in tasks such as object recognition, image segmentation, facial recognition, and more due to their ability to extract meaningful features from images.

**Figure 7:**
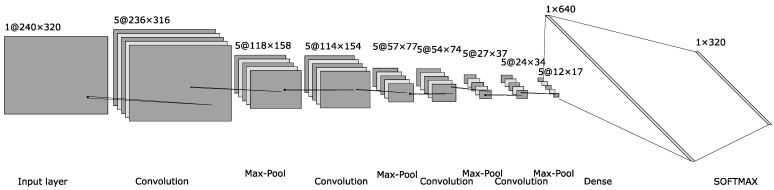
The architecture of the neural network classifier used to detect the *x-coordinate* of the pupil center.

The CNN architecture used in this application for detecting the *x-coordinate* of the pupil center is illustrated in Figure 7 and includes the following layers:

- The input layer of the network has 320 × 240 neurons that encode the normalized light intensities of the pixels of the image to be processed.
- Layer 2, the first hidden layer, is a convolutional layer that uses a 5-by-5 receptive field and 5 feature maps. This results in a layer of 5 × 316 × 236 hidden feature neurons.
- The 3rd layer is one of max-pooling, with the role of simplifying the information, which is applied to the 2-by-2-size regions of each of the 5 feature maps. This results in a layer of hidden feature neurons of size 5 × 158 × 118.
- A new convolutional layer follows with the same size for receptive fields, leading to a hidden feature layer of size 5 × 154 × 114, followed by a max-pooling layer, leading to size 5 × 77 × 57.
- After the third convolution on a 4-by-4 receptive field and the third 2-by-2 max-pooling operation, a layer of hidden feature neurons of size 5 × 37 × 27 results.
- The last convolution layer is applied on a receptive field of 4 by 4, and after the last max-pooling operation, a layer of 5 × 17 × 12 neurons results.
- The penultimate layer is a fully connected one with 640 neurons.
- The last layer is a SOFTMAX type and has a size equal to the horizontal size of the input image.

The final layer of classifiers implemented with neural networks, which uses the SOFTMAX function, is essential for transforming the final network scores or activations into probability distributions. This is important in classification problems for which we want to assign different classes to some input data because the SOFTMAX function transforms the final network scores or activations into a probability distribution so that the sum of the probabilities for all classes equals 1. This makes it possible to interpret the results as class probabilities. After applying the SOFTMAX function, we can select the class with the highest probability as the final prediction of the network for a given input example. This makes the results easier to interpret and use in practical applications. In many classification problems, the cost function (e.g., cross-entropy) is based on the probabilities predicted by the network. Applying the SOFTMAX function facilitates the calculation of this cost function because it transforms the scores into probabilities. Also, the SOFTMAX function has an implicit regularization property, as it penalizes overconfident predictions. This can help prevent overtraining in certain cases. Moreover, it is compatible with gradient-based optimization algorithms such as stochastic gradient descent (SGD) and its variants. This makes training SOFTMAX models easier and more efficient.

Thus, the SOFTMAX function is an essential element in the final layer of neural network-based classifiers because it transforms the scores into probability distributions that are easy to interpret and can be used in a wide range of classification applications.

**Figure 8:**
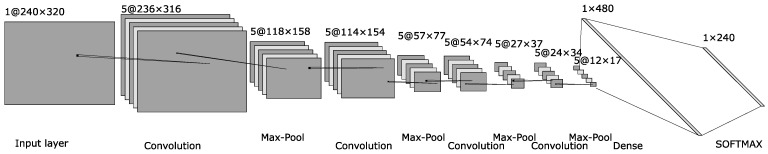
The architecture of the neural network classifier used to detect the *y-coordinate* of the pupil center.

The architecture of the classifier responsible for detecting the *y-coordinate* of the pupil center is illustrated in Figure 8. The only differences compared to the classifier for the *x-coordinate* are in the dense layer and in the SOFTMAX layer: the dense layer has 480 neurons, and the SOFTMAX output layer has 240 neurons, representing the vertical dimension of the input image.

To evaluate the total number of parameters in this neural network, we need to consider all layers and calculate the number of parameters for each layer separately. Network parameters include weights and biases for each layer, resulting in 858,990 and 695,150 trainable parameters for *X*—Classifier and *Y*—Classifier, respectively.

## 3. Training and Results

**Figure 9:**
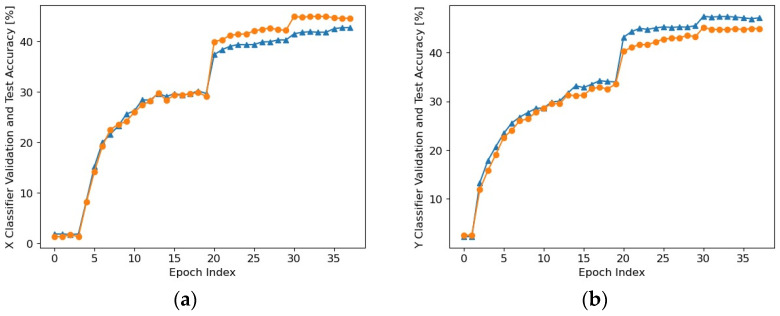
Accuracy on validation data (Δ) and test data (o) during the training of the *X* Classifier (**a**) and *Y* Classifier (**b**).

The implementation of the proposed pupil detection system based on neural network classifiers suitable for high-resolution eye images was performed in python using as a starting point the environment developed in [35]. The two neural networks that implement the classifiers for the *x-coordinate* and *y-coordinate*, respectively, were trained in a similar way to the network presented in Section 2.1. Stochastic gradient descent was used, starting with a high learning rate (*η*) for a relatively large number of epochs in a randomly initialized network and then continuing with lower learning rates in a smaller number of epochs, as Figure 9 illustrates (*η* = 0.1 for 20 epochs, *η* = 0.01 for 10 epochs, *η* = 0.001 for 5 epochs, and then *η* = 0.0001 for 3 epochs), the network being pretrained in the previous steps. In order to reduce overfitting, a weight decay technique was used, with the regularization parameter being *λ* = 0.1. In this way, a trade-off was made between finding small weights and minimizing the cost function. 80% of the total number of available images constituted the train set, 10% of the total images constituted the test set, and the remaining 10% represented the validation set.

The end of training indicated a detection accuracy of approximately 45% for both the *Y* Classifier and the *X* Classifier, meaning that the pupil center coordinates, separately, were detected with an error of 0 pixels for almost half of the images. However, pupil center detection with an error of less than 10 pixels can be considered a useful result. Consequently, in order to form a clear picture of the effectiveness of this classifier, we resorted to the feedforward processing (inference) of each of the images included in the 20 databases considered (our own database [7], a database of images from Casia Iris Lamp [32], the Świrski database [33], and 17 databases provided by the authors of the ExCuSe algorithm [34]).

**Figure 10:**
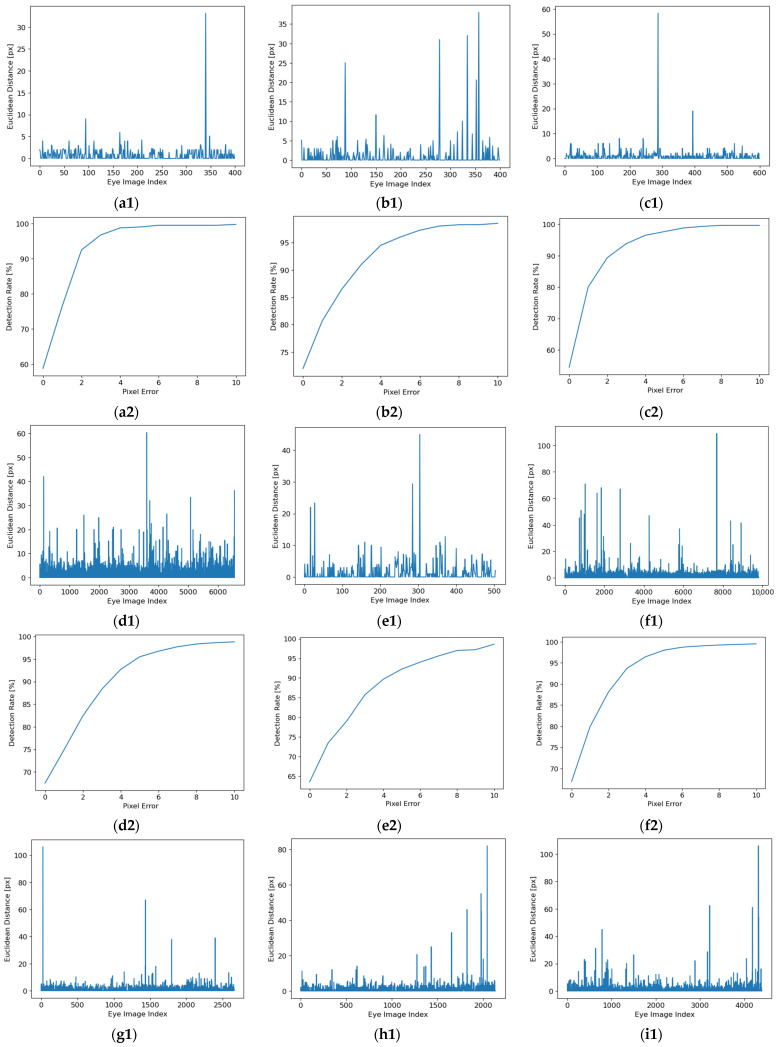

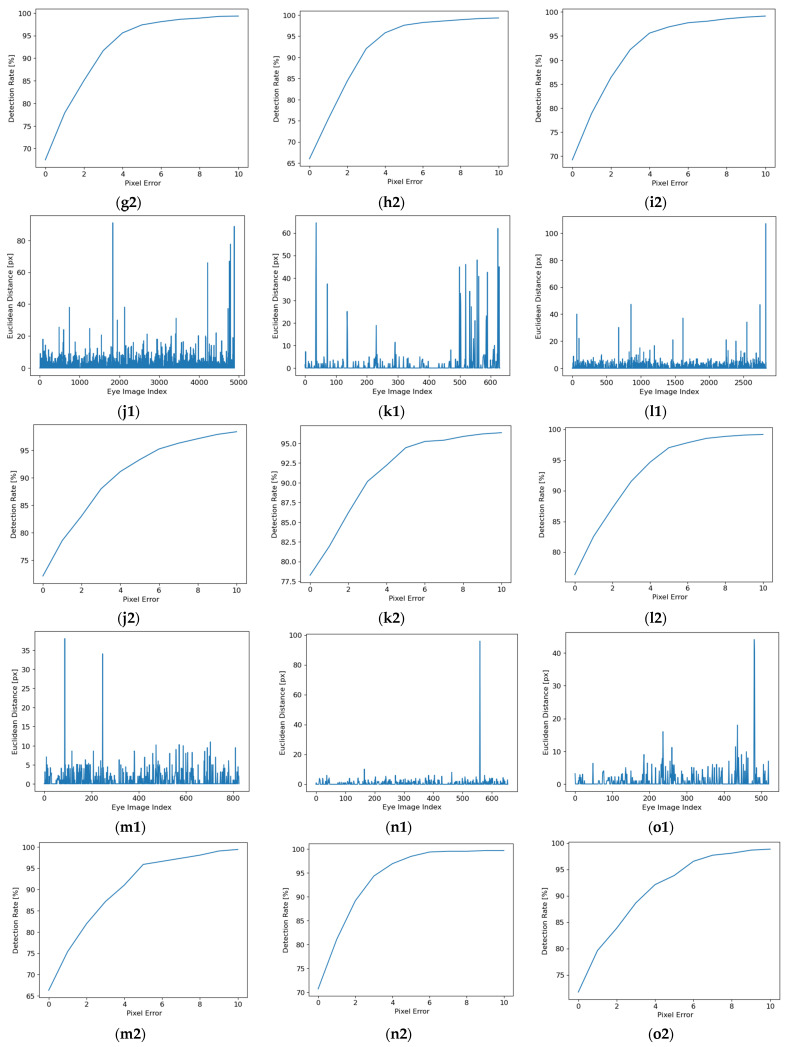

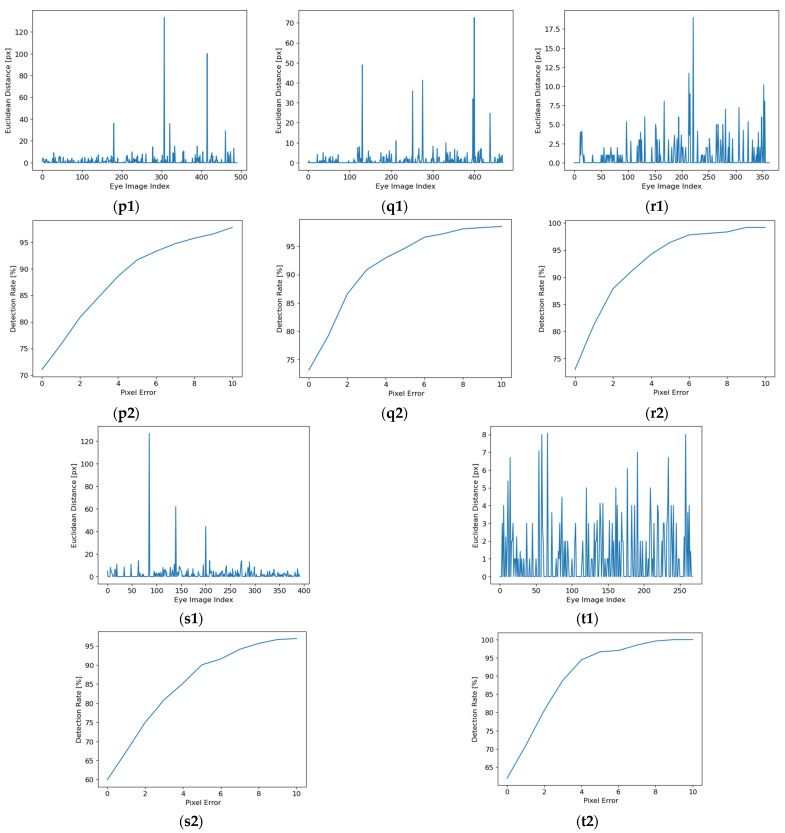
The Euclidian distance (between the detected and the manually labeled pupil center) for each eye image from each database: (**a1**) DB1; (**b1**) DB2; (**c1**) DB3; (**d1**) DSI; (**e1**) DSII; (**f1**) DSIII; (**g1**) DSIV; (**h1**) DSV; (**i1**) DSVI; (**j1**) DSVII; (**k1**) DSVIII; (**l1**) DSIX; (**m1**) DSX; (**n1**) DSXI; (**o1**) DSXII; (**p1**) DSXIII; (**q1**) DSXIV; (**r1**) DSXV; (**s1**) DSXVI; (**t1**) DSXVII. The detection rate depends on pixel error for all eye images from each database: (**a2**) DB1; (**b2**) DB2; (**c2**) DB3; (**d2**) DSI; (**e2**) DSII; (**f2**) DSIII; (**g2**) DSIV; (**h2**) DSV; (**i2**) DSVI; (**j2**) DSVII; (**k2**) DSVIII; (**l2**) DSIX; (**m2**) DSX; (**n2**) DSXI; (**o2**) DSXII; (**p2**) DSXIII; (**q2**) DSXIV; (**r2**) DSXV; (**s2**) DSXVI; (**t2**) DSXVII.

In the following, the results of inference for each database are presented in Figure 10.

In order to evaluate the accuracy of the proposed solution, we used pixel error, representing the Euclidean distance between the detected and manually labeled pupil centers, given by the following equation:

(3) $$d(c_{ideal},\;c_{detected})=\left|c_{ideal}-c_{detected}\right|$$

where $c_{ideal}$ and $c_{detected}$ represent the coordinates of the ideal pupil center (manually labeled) and the one detected by the proposed solution, respectively.

In Figure 10, the Euclidean distance (between the detected and the manually labeled pupil center) for each eye image from each database and the detection rate (DR) depending on pixel error for all eye images from each database are presented. In these figures, the detection rate is calculated for values of the Euclidean distance between 0 and 10 pixels, but the performance of the proposed solution is estimated at 5 pixels.

The detection rate at *k* pixels ($DR_{k}$) represents the ratio between the number of images for which the Euclidean distance between the center of the pupil detected by the proposed solution and the manually labeled one is less than or equal to *k* pixels ($N_{k\;px}$) and the total number of images in the database ($N_{total})$.

(4) $$DR_{k}=\frac{N_{k\;px}}{N_{total}}\cdot 100\;(\%)$$

The experimental results illustrated in Figure 10 show a detection rate of 5 pixels between 90.05% and 99% and a standard deviation between 1.71 pixels and 8.22 pixels for all eye images from each database tested.

**Figure 11:**
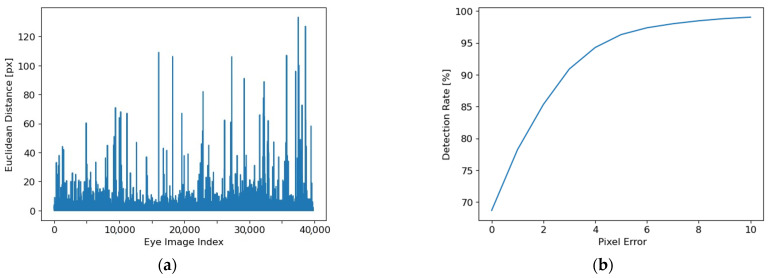
Results of the inference for all 20 databases: (**a**) Euclidian distances for each of the ~40 k images; (**b**) detection rates depending on pixel error for the eye images in all databases.

The systematized results for the entire database (including all 20 eye image databases) are illustrated in Figure 11.

For all databases, the 5-pixel detection rate is DR5 = 96.29%, while the dispersion is SIGMA = 3.38 pixels. The average time required for the feedforward processing of an image is 10 ms, which means a framerate of 100 frames/s.

The high accuracy values obtained on a large number of images taken from different sources, of great diversity, and acquired under variable and non-uniform lighting conditions, including numerous reflections and obstructions, prove the robustness of the proposed solution. In addition, the small values of SIGMA guarantee the stability of the cursor on the user’s screen for real-time applications.

All these results prove the performance of the proposed solution (in terms of accuracy, running time, and robustness) on large databases that include eye images of great diversity, acquired in different difficult lighting conditions, specific to real-time applications.

## 4. Discussion

**Figure 12:**
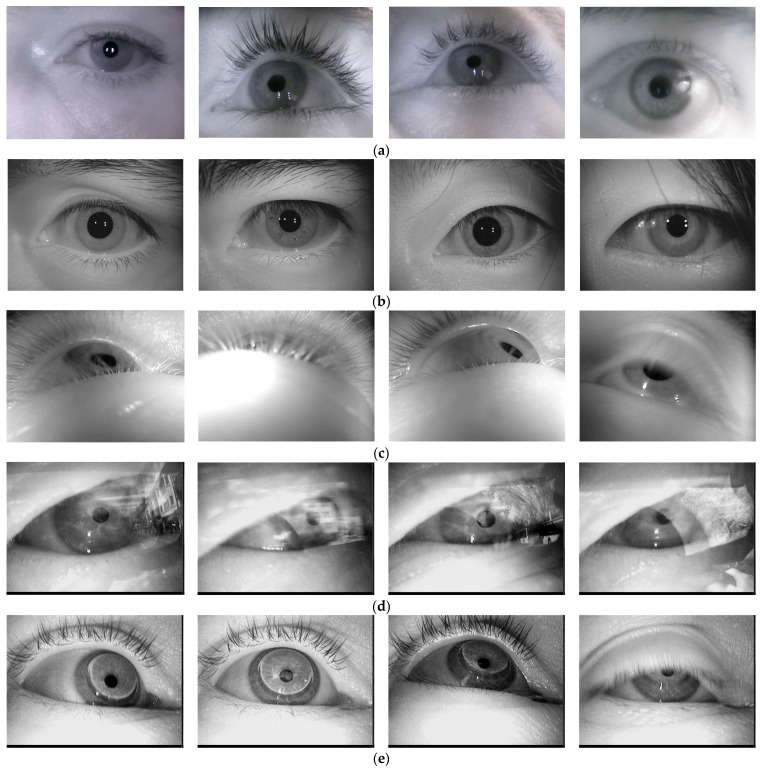

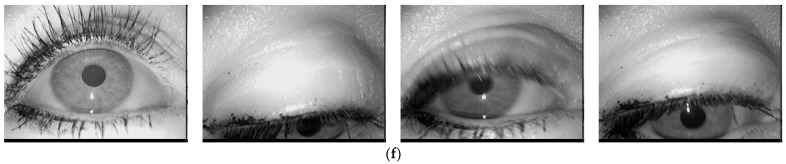
Samples of the eye images used in our study from different databases: (**a**) DB1; (**b**) DB2; (**c**) DB3—Świrski database; (**d**) DSI—with reflections; (**e**) DSIV—with contact lenses, bad illumination; (**f**) DSXVI—with mascara, eyelashes.

The performance of the proposed AI-based pupil detection system, in terms of detection accuracy, was tested on ~40,000 eye images taken from 20 different databases available in the literature [7,32,33,34]. In Figure 12, some representative eye images from these databases are illustrated.

In order to be processed by the proposed neural network, all the images in these databases were resized to a resolution of 320 × 240 pixels, considered suitable for real-time applications, in which the eye images are captured with remote-type eye tracking interfaces.

Due to the availability of test results for the same databases, the accuracy of pupil detection for the proposed solution was compared with that of three algorithms based on classical image processing methods and with a pupil detection method based on CNNs that utilized different approaches. The detection rate at 5 pixels of the proposed solution (XY-CLS) is illustrated in Table 1 and is compared with that of the ExCuSe [8], ElSe [9], and Świrski [33] algorithms (which are based on classical eye image processing), as well as PupilNet v2.0 [14] (implemented by using a dual convolutional neural network pipeline for image-based pupil detection). PupilNet v2.0 detects the pupil center in several stages: the first stage, implemented with a CNN, infers a coarse estimate of the pupil location. The second stage refines the initial estimate by using a second CNN. The results provided by PupilNet v2.0 are obtained through different approaches: direct approach (*SK*_8_*P*_8_)–direct coarse positioning and fine positioning, implemented with $F_{CK_{x}P_{y}}$ and $F_{SK_{x}P_{y}}$. Also, Table 1 presents the standard deviation of the Euclidean distance obtained by the proposed implementation for each of the 20 databases considered, as well as a short description of the conditions in which the eye images were obtained.

According to the results presented in Table 1, first of all, it is observed that the proposed solution shows significantly better results when applied to databases with images of the eye acquired in bad illumination, such as DSII, DSIII, DSIV, DSVI, DSVII, DSX, DSXI, DSXII, and DSXIII. It is worth noting that for the DSXVII database (with a small number of images), the PupilNet v2.0 solution illustrated in [14] shows a higher accuracy at 5 pixels than our solution. The differences are very small and can be corrected in future implementations by increasing the size and diversity of the eye image databases used to train the network.

For images with reflections, specific to real-time applications or taken through glasses (such as those in DSI, DSII, DSIII, DSIX and DSXI), the proposed method also shows better results than the other algorithms in Table 1.

The proposed architecture also shows better results for images of the eye taken through contact lenses, as observed for the databases DSIV, DSV, and DSXV.

It is also observed that the proposed solution shows better results for eye images that contain objects with gray intensity levels similar to the pupil, such as mascara, eyeshadow, and eyelashes, which are specific to DSVI, DSVII, DSVIII, DSXIII, and DSXVI. Such images.

(Containing objects with pupil-like gray intensity levels) are very common in eye image databases, since most interfaces used for gaze detection use infrared video cameras for eye image acquisition. For such images, most of the classic algorithms that are based on the dark pupil technique [1] produce significant errors, as observed for the databases mentioned above as well as for many other similar situations illustrated in the literature [7].

The proposed method for pupil center detection also provides significantly better results for eye images acquired from different individuals (such as the Iris Casia Lamp database) or with different geometries (circles or ellipses with different orientations, due to the different angles at which the eyes were filmed), as well as for images of the eye with the pupil partially obscured by eyelashes or eyebrows, such as the images from the Świrski database [33], as shown in Figure 12. Also, the proposed solution shows very good results for eye images with multiple corneal reflections, as is the case with the images from the DB1 and DB2 databases.

In most cases, algorithms based on classical image processing provide good results for eye images taken with a certain type of eye tracking interface and under the same lighting conditions, but they produce very poor results for other types of interfaces, as shown in [7] for the Starburst and ExCuSe algorithms tested on databases DB1 and DB2, respectively. It is worth noting that the proposed solution provides very good results for eye images acquired with different eye tracking interfaces, such as those used for acquiring images from DB1, DB2, DB3, and DSI-DSXVII, respectively.

The results presented in Table 1 indicate that the proposed solution exhibits a good detection rate under conditions of variable illumination of the eye; in scenarios such as the presence of reflections, poor lighting, and glasses or contact lenses; the intrusion of eyelashes or mascara; and corneal reflections. The results we obtained are significantly better than those published for algorithms based on classical image processing, confirming the expected robustness characteristic of technologies based on artificial intelligence.

The reduced value of the standard deviation, calculated for the Euclidean distance between the ideal center (manually labeled) of the pupil and the center detected by the proposed solution, significantly improves the stability of the cursor position on the user’s screen in the case of real-time applications, such as computer control by gaze detection.

There are a small number of lost frames (images of the eye for which the Euclidean distance is greater than 10 pixels). The technical solution to increase the reliability of the system is to implement an algorithm that, for real-time operation, considers the position of the center of the pupil in several consecutive frames, to extract either an average of the detected coordinates or to eliminate the coordinates that differ too much from those detected in the preceding/succeeding frames.

The performance of the proposed AI-based architecture depends on the size and diversity of the databases used for training, which must include eye images acquired in different lighting conditions by using different eye tracking interfaces. The large size of the eye image databases makes it difficult to build ground truth data, which includes hand-labelled eye images.

The good detection performance of the presented technical solution suggests the system should be recommended for all real-time gaze tracking applications, but especially in applications characterized by variable eye illumination, such as driver monitoring.

The proposed solution was compared in terms of the number of trainable parameters of the neural network with other AI-based implementations [30,36,37,38,39,40,41,42,43,44,45,46] for the same purpose, as depicted in Table 2. The smallest number of parameters is necessary to increase the computational efficiency, to increase the generalization capacity of the model, to prevent overfitting, and to increase the interpretability of the model, respectively. The proposed solution stands out for its reduced number of parameters, especially if you individually compare the two classifiers that each give one coordinate of the center of the pupil. Moreover, our system provides the pupil center coordinates, while the other AI-based architectures illustrated in the literature [36,47] provide only one of a limited number of pupil positions.

The uniformity of the results obtained in terms of detection accuracy at 5 pixels (over 90% for all the 20 databases with eye images of great diversity) proves the robustness and reliability of the proposed solution.

A possible improvement to our solution is the addition of an output dedicated to the case when there is no pupil in the image. The training/testing/validation dataset must be supplemented with a significant number of images in which there is no pupil in order to improve the reliability of the algorithm.

In a future paper, this AI-based pupil detection system will be used to implement different real-time applications in the field of assistive technology in order to communicate with neuromotor-disabled patients by using keyword technology or in the automotive industry to increase driving safety by detecting driver distraction and recognizing driver drowsiness based on eye tracking technology. Also, due to the complexity of the eye image databases used for training, the proposed algorithm could be used for estimating perceived cognitive load based on eye tracking technology. In order to execute this, the neural network will provide both the center of the pupil and its area, whose dynamic variation over time will provide information on the cognitive state of the subject. Using several parameters provided by the neural network and monitoring their dynamic variation over time, it will be possible to estimate the perceived mental workload related to a wide range of types of cognition, such as attention, memory, problem solving, evaluation, reasoning, understanding, and language organization.

## 5. Conclusions

In this paper, new AI-based architectures for pupil center detection using neural network classifiers capable of processing low- and high-resolution eye images have been presented.

In order to reduce complexity and processing time, the proposed AI-based pupil detection system includes two slim neural networks that process the information in parallel to provide the two pupil center coordinates on the *x* and *y* axes. Therefore, the proposed architecture includes a significantly smaller number of parameters than other types of neural networks published in the literature.

The proposed pupil detection system was tested on ~40,000 eye images from 20 different databases, resized to a resolution of 320 × 240 pixels, that include images acquired from different individuals in variable and non-uniform lighting conditions, having different shapes, with different reflections, covered of eyelashes and eyebrows, using mascara or contact lenses.

The experimental results showed a detection rate of 5 pixels between 90.05% and 99% and a standard deviation between 1.71 pixels and 8.22 pixels for all eye image databases tested. For the entire database (which includes all 20 eye image databases), the 5-pixel detection rate is DR5 = 96.29%, while the dispersion is SIGMA = 3.38 pixels. The average time required for the feedforward processing of an image is 10 ms, which means a framerate of 100 frames/s, which is suitable for real-time applications.

Unlike PDAs based on classical image processing, AI-based solutions significantly increase the diversity of eye images as well as the types of interfaces used for gaze detection for which they work properly. The performance of these systems depends on the number and diversity of the eye image databases that were used to train the neural network.

According to these results, the proposed solution is suitable for processing high-resolution eye images in real-time systems due to its high accuracy and reduced processing times. As expected, the detection accuracy is significantly better than in the case of non-AI detection algorithms due to their good immunity to variable lighting conditions or the presence of partial obstructions.

## 6. Patents

Application: A/00586 20.10.2023—Method for reducing the costs of implementing neural network technology-based classifiers used in gaze detection.

## Figures and Tables

**Table 1:** Results obtained by the proposed solution in comparison with those of the ExCuSe [9], ELSE [10], Świrski [33], and PupilNet v2.0 [14] approaches.

Data Base	Frames Number	DR5 [%]							Standard Deviation [pixels]	Description
		ExCuSe	ElSe	Świrski	PupilNet v 2.0			XY-CLS		
					$SK_{8}P_{8}$	$F_{CK_{x}P_{y}}$	$F_{SK_{x}P_{y}}$			
DB1	400	82.25	-	-	-	-	-	99	1.95	Local device captures
DB2	400	84.25	-	-	-	-	-	96	3.58	Casia Iris Lamp [32]
DB3	600	86.17	82.00	77.17	-	-	-	97.66	2.77	Świrski database [33]
DSI	6554	70.95	85.52	5.11	77	78	82	95.51	2.59	Reflections
DSII	505	34.26	65.35	26.34	80	79	79	92.27	3.41	Reflections, bad illumination
DSIII	9799	39.44	63.60	6.81	62	60	66	97.97	2.73	Reflections, recording errors, bad illumination
DSIV	2655	81.58	83.24	34.54	90	90	92	97.36	3.37	Contact lenses, bad illumination
DSV	2135	77.28	84.87	77.85	91	89	92	97.56	3.15	Shifted contact lenses
DSVI	4400	53.18	77.52	19.34	73	78	79	96.88	3.1	Bad illumination, mascara
DSVII	4890	46.91	59.51	39.35	73	80	73	93.29	3.82	Bad illumination, mascara, eyeshadow
DSVIII	630	56.83	68.41	41.90	84	83	81	94.44	6.83	Bad illumination, eyelashes
DSIX	2831	74.96	86.72	24.09	86	86	86	97.03	3.24	Reflections, additional black dot
DSX	840	79.76	78.93	29.88	80	78	81	95.87	2.58	Bad illumination, pupil at image boarder
DSXI	655	56.49	75.27	20.31	85	74	91	98.47	3.94	Reflections, bad illumination, additional black dot
DSXII	524	79.20	79.39	71.37	87	85	85	93.85	3.3	Bad illumination
DSXIII	491	70.26	73.52	61.51	79	81	83	91.64	8.22	Bad illumination, eyelashes
DSXIV	469	57.57	84.22	53.30	91	94	95	94.66	5.28	Bad illumination
DSXV	363	52.34	57.30	60.88	81	71	81	96.41	1.94	Shifted contact lenses
DSXVI	392	49.49	59.95	17.86	80	72	80	90.05	7.81	Mascara, eyelashes
DSXVII	268	77.99	89.55	70.90	99	87	97	96.64	1.71	Bad illumination, eyelashes

**Table 2:** A comparison of the number of trainable parameters between the proposed system and similar AI-based pupil detection techniques.

Model	Number of Trainable Parameters
LeNet5 [37]	1,742,431
AlexNet [30]	15,906,511
DenseNet [38]	9,860,431
ResNet [39]	42,530,895
Vgg16 [40]	15,120,975
VIT [41]	1,073,485,056
Convnext [42]	27,810,255
Swin-T [43]	28,265,032
SqueezeNet [44]	1,248,424
MobileNet [45]	3,222,351
Shufflenet [46]	911,529
PONet [36]	4,950,671
*X*—Classifier—proposed in this paper	858,990
*Y*—Classifier—proposed in this paper	695,150

## Data Availability

Data are contained within the article.
